# A comparative study on mental health outcomes in patients with rheumatoid arthritis and knee osteoarthritis

**DOI:** 10.3389/fmed.2026.1879435

**Published:** 2026-09-03

**Authors:** Meng Jia, Shuang Wang, Chenxi Yu, Ping Li

**Affiliations:** Shandong Provincial Hospital, Jinan, China

**Keywords:** comparative study, mental health, osteoarthritis, rheumatoid arthritis, SCL-90

## Abstract

**Objective:**

This study aimed to analyze the current status and differences in mental health levels between patients with rheumatoid arthritis (RA) and knee osteoarthritis (OA) to provide a reference for clinical nursing interventions.

**Methods:**

Using convenience sampling, patients with knee osteoarthritis and rheumatoid arthritis who were hospitalized at a tertiary first-class hospital in Shandong Province from May to August 2025 were selected for a cross-sectional survey, and the differences in mental health levels between the two groups were compared.

**Results:**

There were significant differences between the OA and RA groups in the Symptom Checklist-90 (SCL-90). Except for terror, paranoia, and other dimensions, there were statistically significant differences in the scores for each SCL-90 dimension between the two groups (*p* < 0.001).

**Conclusion:**

In summary, RA patients exhibit lower psychological health levels than OA patients. In addition, there were differences in social demographic characteristics that affected the mental health of both groups. In clinical practice, healthcare professionals should focus on mental health of RA and OA patients, acknowledge the adverse consequences of psychological problems, and carry out targeted interventions to improve patients’ quality of life and their mental wellbeing.

## Introduction

Arthritis is a common chronic disease among the elderly. The most common types of arthritis are knee osteoarthritis (OA) and rheumatoid arthritis (RA). According to WHO reports (35), as of 2020, the global number of OA patients had increased to 595 million, representing a 132% increase. The prevalence of RA is approximately 0.27–0.46% (1, 2), indicating an increasing trend year by year. The main clinical symptoms of both conditions are pain, swelling, joint dysfunction, and deformity at the joint site. In severe cases, these symptoms can lead to disability. Patients often endure prolonged physical pain, gradually lose their ability to work, and face significant mental stress due to additional treatment costs, which can easily lead to serious psychological problems (3). According to previous research (4, 5), anxiety, depression, and other adverse psychological conditions in OA and RA patients are more prevalent than the general population. Current research mainly focuses on the comparison of health-related quality of life between the two patient groups.

The majority of comparisons regarding health-related quality of life issues focus on the physical functioning of patients with OA and RA (6). This includes examining improvements in functional outcomes after total knee replacement in both groups (7). However, relatively few studies address the mental health of patients with OA and RA. Some studies have pointed out that all patients with RA have mental health problems due to role limitations caused by their physical conditions, which are mainly related to depression and anxiety. However, compared to OA patients, RA patients have lower psychological expectations due to long-term limitations in functional activities and persistent pain (8). The lower satisfaction levels of RA patients (9) also suggest that improving their mental health and enhancing their overall satisfaction has become an important part of clinical work.

To the best of our knowledge, only two studies have compared mental health between patients with RA and OA. A 2007 study showed (10) that anxiety and depression in patients with RA and OA severely affect their quality of life, but it did not clarify the differences in anxiety and depression between the two groups of patients. Another recent study (11) pointed out that the application of the Patient-Reported Outcomes Measurement Information System scale can effectively assess mental health problems in patients with musculoskeletal diseases; however, the study did not report the analysis of results comparing mental health between the two patient groups.

Although previous findings on psychological characteristics indicate that the incidence of negative emotions, such as depression and anxiety, in RA patients is higher than that in OA patients (6, 12), the differential characteristics and influencing factors of mental health between the two types of patients have not been fully verified. Therefore, this study aims to investigate the mental health levels of rheumatoid arthritis and knee osteoarthritis patients and compare the differences and influencing factors affecting mental health between the two types of patients.

## Materials and methods

### Clinical sample

This observational, cross-sectional study investigated the differences in mental health levels between patients with RA and those with OA. The sample consisted of patients aged 18 years or older diagnosed with RA according to the ACR/EULAR classification criteria (13) and with OA based on the Chinese guidelines for diagnosis and treatment of osteoarthritis (2024 edition) (14). Participants included in this study were diagnosed with OA and RA at a tertiary hospital in Shandong Province from May to August 2025, and informed consent was obtained from all subjects. The exclusion criteria were as follows: presence of serious cognitive impairment or mental illness; presence of other serious diseases (such as severe infections, heart disease, respiratory disease, and kidney disease) that could affect disease activity. The study was approved by the ethics committee of Shandong First Medical University (No. R202311090192). All research procedures were carried out in accordance with the Declaration of Helsinki and the guidelines for Good Clinical Practice. Informed consent was obtained from all participants involved in this study.

### Measurements

(1) A self-made general information form was used to collect basic clinical characteristics of the two groups, including age, gender, marital status, and number of comorbidities.
(2) The Symptom Checklist-90 (SCL-90) is a widely recognized measurement tool for evaluating mental health. It is mainly used for interpersonal sensitivity screening to identify whether individuals have psychological problems and determine psychological symptoms (15). This study used the SCL-90 tool to investigate the mental health status of patients with OA and RA. Developed by Derogatis, it includes nine subscales: somatization, obsessive-compulsive symptoms, interpersonal sensitivity, depression, anxiety, hostility, terror, paranoia, and psychosis, with a total of 90 items. A 5-point Likert scoring method was used. The positive detection criteria for screening are as follows: a total score of ≥160 points, an average score of ≥3 points on any factor, or more than 43 positive items.
(3) The Self-Regulatory Fatigue Scale (SRF-S) was developed by Nes et al. (16) based on existing research and is used to assess the psychological ego-depletion state of patients. In this study, the Chinese version of the Self-Regulatory Fatigue Scale, translated by Wang (17), is used to investigate the self-regulatory fatigue state in RA and OA patients. This scale includes three dimensions: cognitive control, emotional control, and behavioral control. There are 16 items in total. A 5-point Likert (1–5 points) scoring method is adopted. The higher the score, the more serious the self-regulatory fatigue.
(4) The Acceptance and Action Questionnaire-II(AAQ-II) was developed by foreign scholars, including Livheim, and later adapted and revised by domestic scholars, such as Cao Jing. This questionnaire assesses an individual’s experiential avoidance level. The scale consists of 7 items, each scored on a 7-point Likert scale, ranging from ‘never’ to‘always,’ with scores of 1 to 7. The total score ranges from 7 to 49, with higher scores indicating higher experiential avoidance and lower psychological flexibility.

### Statistical analysis

Variables were described as absolute frequencies and percentages for categorical data, while continuous variables were presented as means (SDs). Categorical variables were compared between the two groups using the chi-squared test, and continuous variables were compared using t-tests or ANOVAs with Bonferroni’s post-hoc comparisons. To explore the correlation between each characteristic (independent variable) and the total score and each dimension of the SCL-90, univariate linear regression analysis was performed. For comparisons involving variables with three or more groups (e.g., course of disease, educational level, economy, and comorbidities), one-way analysis of variance (ANOVA) was performed, followed by Bonferroni post-hoc tests for pairwise comparisons to control the family-wise error rate. The Bonferroni-corrected significance threshold for post-hoc comparisons was set at*α* = 0.05 divided by the number of pairwise comparisons within each ANOVA. Spearman’s correlation analysis was performed to examine the correlations between SCL-90, AAQ-II, and SRF-S. Multiple linear regression analysis was performed to examine the factors influencing mental health in the two groups. All tests were bilateral, with a significance level of *p* < 0.05. All analyses were performed using SPSS (version 26) for Windows.

## Results

A total of 269 patients were included in our study, comprising RA patients (*n* = 106, 39.4%) and OA patients (*n* = 163, 60.6%). A post-hoc power analysis confirmed that our sample of 269 participants is sufficient for detecting a medium effect size with 5 predictors at *α* = 0.05 and power = 0.80. There were no statistically significant differences in any sociodemographic or clinical characteristics, except for age and disease course (Table 1).

**Table 1:** Sociodemographic and clinical characteristics (n = 269).

Sociodemographic characteristics	RA (106)		OA (163)		t/F	*p*-value
	*N*	%	*N*	%		
Age, years(±s)	51.84 ± 11.74		62.12 ± 7.96		8.563	<0.001^a^
Gender						
Men	58	54.7	82	50.3	0.501	0.479^b^
Women	48	45.3	81	49.7		
Disease duration						
<5 years	14	13.2	2	1.2	1.856	<0.001^b^
5 ~ 9 years	70	66.0	110	67.5		
≥10 years	22	20.8	51	31.3		
Comorbidities, kinds						
0	22	20.8	25	15.4	5.277	0.071^b^
1 ~ 2	44	41.5	91	55.8		
≥3	40	37.7	47	28.8		
Marital status						
Single	39	36.8	55	33.7	0.263	0.608^b^
Married	67	63.2	108	66.3		
Educational level						
Primary	35	33.0	57	35.0	1.784	0.618^b^
Secondary	40.6	43	74	45.4		
Tertiary	22.6	24	28	17.2		
Other	4	3.8	4	2.4		
Economy (Yuan/month)						
<3,000	41	38.7	57	35.0	1.172	0.557^b^
3,000 ~ 5,000	49	46.2	86	52.8		
≥5,000	16	15.1	20	12.2		

RA, rheumatoid arthritis; OA, knee osteoarthritis. ^a^ANOVA; ^b^chi-square test.

The majority of RA patients were men (54.7%), married (63.2%), and had a secondary educational level (40.6%). The RA group was younger (mean 51.84 years, SD 11.74) and had 1–2 types of comorbidities (41.5%). In contrast, OA patients were older (mean 62.12, SD 7.96). Approximately 79.2% of RA patients and 84.6% of OA patients had comorbidities. The disease duration was also longer in the OA group than in the RA group.

There were significant differences between the two groups in the AAQ-II, SRF-S, and SCL-90 scores (*p* < 0.001). Apart from the terror and paranoia dimensions, all other SCL-90 dimension scores showed statistical differences between the two groups (*p* < 0.001; Table 2).

**Table 2:** Comparison of AAQ-II, SRF-S, and SCL-90 scores between the two groups.

Variables	RA (*n* = 106)		OA (*n* = 163)		*t*	*p*-value
	Mean	SD	Mean	SD		
AAQ-II	30.44	11.11	14.40	6.72	−14.750	<0.001
SRF-S	50.93	6.92	42.17	6.17	−10.848	<0.001
SCL-90						
Total score	151.99	7.70	115.22	6.81	−42.629	<0.001
Somatization	25.06	2.89	17.77	4.09	−15.967	<0.001
Obsessive-compulsive symptoms	27.05	4.26	11.72	1.27	−43.143	<0.001
Interpersonal sensitivity	20.45	4.47	14.25	2.64	−14.314	<0.001
Depression	20.57	3.84	16.47	3.13	−9.575	<0.001
Anxiety	18.36	3.08	15.36	3.14	−7.724	<0.001
Hostility	7.19	1.69	6.61	0.76	−3.791	<0.001
Terror	7.36	0.91	7.46	0.71	1.030	0.304
Paranoia	6.56	1.12	6.64	0.92	0.652	0.515
Psychosis	10.46	1.13	9.96	0.83	−4.226	<0.001
Other	8.94	1.94	8.98	1.19	0.200	0.842

RA, rheumatoid arthritis; OA, knee osteoarthritis.

The total scores of the SCL-90, AAQ-II, and SRF-S for the two groups were analyzed using an univariate analysis. Age, gender, disease duration, marital status, educational level, economic status, and number of comorbidities were treated as independent variables, and the total scores of SCL-90, AAQ-II, and SRF-S were treated as dependent variables sequentially. A univariate analysis was conducted using t-test or ANOVA. The results showed that in the OA group, age, gender, disease duration, marital status, educational level, economic status, and number of comorbidities had an impact on the total SCL-90 score, and the differences were statistically significant (*P<*0.05). Bonferroni post-hoc tests further showed that patients with 1–2 comorbidities [mean difference (MD) = 6.545, SE = 0.795, *p* < 0.001] and those with≥3 comorbidities (MD = 14.542, SE = 1.025, *p* < 0.001) had significantly higher SCL-90 scores compared to those without comorbidities (see Supplementary Table S1 for detailed post-hoc results). For AAQ-II scores, significant differences were observed across comorbidity categories (*F* = 4.605, *p* < 0.001). For SRF-S scores, gender (*t* = 2.540, *p* = 0.012), marital status (t = −2.905, *p =* 0.004), disease duration(*F* = 3.051, *p* = 0.032), economic status (*F* = 3.900, *p* = 0.022), and comorbidities (*F* = 26.870, *p* < 0.001) showed significant associations.

In the RA group, age (*F* = 1.703, *p* = 0.033), gender (*t* = −8.503, *p* < 0.001), and course of disease (*F* = 40.005, *p* < 0.001) had a significant impact on SCL-90 scores. Bonferroni post-hoc tests for disease duration revealed that patients with 5–9 years (mean difference = 4.693, SE = 1.134, *p* < 0.001) and≥10 years (MD = 9.024, SE = 1.652, *p* < 0.001) had significantly higher SCL-90 scores than those with <5 years. For AAQ-II scores, marital status (*t* = −2.197, *p* = 0.030) and comorbidities (*F* = 4.094, *p* = 0.019) showed significant associations. For SRF-S scores, comorbidities (*F* = 4.115, *p* = 0.019) was the only significant factor. No significant associations were found for educational level or economic status across any of the three outcome measures in the RA group. (Table 3).

**Table 3:** Total scores of SCL, AAQ-II, and SRF-S of the two groups analyzed by single-factor analysis.

Variables	SCL-90		AAQ-II		SRF-S	
	*t/F*	*p*	*t/F*	*p*	*t/F*	*p*
OA						
Age	1.461	0.080^b^	1.591	0.043^b^	0.883	0.637^b^
Gender	2.942	0.004^b^	1.139	0.256^b^	2.540	0.012^b^
Disease duration	3.698	0.027^a^	0.376	0.688^a^	3.051	0.032^a^
Marital status	−3.331	0.001^b^	0.326	0.745^b^	−2.905	0.004^b^
Educational level	4.655	0.004^a^	0.807	0.492^a^	0.339	0.797^a^
Economy	2.166	0.118^a^	0.673	0.511^a^	3.900	0.022^a^
Comorbidities	194.229	<0.001^a^	4.605	<0.001^a^	26.870	<0.001^a^
RA						
Age	1.703	0.033^b^	1.056	0.413^b^	0.833	0.710^b^
Gender	−8.503	<0.001^b^	−1.715	0.089^b^	−1.473	0.142^b^
Disease duration	40.005	<0.001^a^	1.609	0.205^a^	1.986	0.142^a^
Marital status	−0.392	0.696^b^	−2.197	0.030^b^	−1.007	0.316^b^
Educational level	0.116	0.950^a^	0.112	0.953^a^	0.648	0.586^a^
Economy	0.045	0.956^a^	0.499	0.609^a^	0.544	0.582^a^
Comorbidities	1.215	0.301^a^	4.094	0.019^a^	4.115	0.019^a^

^a^ANOVA; ^b^chi-square test. Significant ANOVA results (*p* < 0.05) were followed by Bonferroni post-hoc tests (see Supplementary Table S1 for detailed post-hoc results). RA, rheumatoid arthritis; OA, knee osteoarthritis. SCL-90: The Symptom Checklist-90; AAQ-II: The Acceptance and Action Questionnaire-II; SRF-S: The Self-Regulatory Fatigue Scale.

Spearman’s correlation analysis showed that the total SCL-90 score was positively correlated with the total AAQ-IIscore and the SRF-S score in the OA group (*p<*0.01), and there was a positive correlation between the total SCL-90 score and both AAQ-IIand the SRF-S scores (*p<*0.01). In RA patients, the SCL-90 score was positively correlated with the total AAQ-IIscore (*p<*0.05), and the total AAQ-IIscore was positively correlated with the total SRF-S score (*p* < 0.01; Table 4).

**Table 4:** Total scores of SCL-90, AAQ-II, and SRF-S compared between the two groups.

Variables	SCL-90	AAQ-II	SRF-S
OA			
SCL-90	1		
AAQ-II	0.283**	1	
SRF-S	0.533**	0.225**	1
RA			
SCL-90	1		
AAQ-II	0.244*	1	
SRF-S	0.140	0.668**	1

RA, rheumatoid arthritis; OA, knee osteoarthritis; SCL-90, The Symptom Checklist-90; AAQ-II: The Acceptance and Action Questionnaire-II; SRF-S: The Self-Regulatory Fatigue Scale. *p<0.05, **p<0.01.

The variables that were statistically significant in univariate and Spearman’s correlation analyses were used as independent variables, and the scores of each scale were used as dependent variables in turn. Multiple linear regression was used to explore the influencing factors of mental health in the two groups.

The study found that female sex, comorbidities, AAQ-II total score, and SRF-S total score were the main influencing factors of the total SCL-90 score in OA patients (see Table 5); female sex, disease duration ≥5 years, AAQ-II, and SRF-S total scores were the main influencing factors of the total SCL-90 score in RA patients (see Table 4). Since the multivariate analysis of the total AAQ-II score and the total SRF-S score in the two groups did not yield specific results, they were not further analyzed in this study.

**Table 5:** Analysis of influencing factors on SCL-90 of OA patients.

Variates	*β*	SE	Beta	*t*	*p*
OA					
Constant	98.632	3.965	-	24.876	<0.001
Female	−1.233	0.564	−0.091	−2.186	0.030
Comorbidities(ref: None)					
1–2	6.545	0.795	0.479	8.230	<0.001
≥3	14.542	1.025	0.971	14.185	<0.001
SRF-S	0.164	0.053	0.148	3.082	0.002
AAQ-II	0.312	0.150	0.316	2.080	0.039
RA					
Constant	146.038	4.437	-	32.914	<0.001
Female	5.898	1.159	0.049	5.090	<0.001
Disease duration (ref: <5 years)					
5 ~ 9 years	4.693	1.134	0.335	4.139	<0.001
≥10 years	9.024	1.652	0.463	5.463	<0.001
SRF-S	0.904	0.101	0.665	8.712	<0.001
AAQ-II	0.495	0.056	0.672	8.903	<0.001

AAQ-II: The Acceptance and Action Questionnaire-II; SRF-S: The Self-Regulatory Fatigue Scale; “-,” means blank space. OA group: R = 0.871, *R*^2^ = 0.758, adjusted *R*^2^ = 0.739; *p*<0.001. RA group: R = 0.761, *R*^2^ = 0.579, adjusted *R*^2^ = 0.553; *p*<0.001.

## Discussion

The results of this survey on the mental health status of OA and RA patients showed that there were significant differences between the two groups in the SCL-90 total score and in the dimensions of somatization, obsessive-compulsive symptoms, interpersonal sensitivity, depression, anxiety, psychosis, and total score. The mental health problems faced by RA patients are generally more severe than those experienced by OA patients. This is closely related to the disease characteristics, disability-causing nature, and long-term treatment burden of RA. Specifically, RA patients often have strong psychological symptoms and perceive somatization symptoms as very obvious, leading to psychological problems that affect their daily lives and work (18). The psychological distress in OA patients is more related to chronic pain and their adaptability to aging (3).

A longitudinal population study in 2010 indicated that both OA and RA patients have a high risk of developing emotional disorders (anxiety and depression), especially younger arthritis patients (age<45 years), who are more prone to it (19). It is estimated that between 14 and 62% of those with RA also suffer from depression (20, 21), significantly higher than OA patients (20–40%). The immune-biological hypothesis states that pro-inflammatory cytokines disrupt the serotonergic system and play a major role in the development of depressive symptoms (22). On the other hand, it may be related to the continuous deterioration and damage of joint function, with the relapse-remitting nature of the disease requiring patients to face the risk of recurrence for a long time (23), resulting in a predisposition to negative emotions in RA patients (24). OA progresses relatively slowly, symptoms are more clearly correlated with activity, and psychological stress is less “unpredictable” (25).

The SCL-90 score in RA patients was also higher than that in OA patients, which may be related to the joint symptoms of RA patients that affect and limit their physical activities, making it difficult for them to engage in normal social activities and causing low self-esteem. The main manifestations include difficulty communicating with others and managing relationships with society, family, and even friends, leading to discomfort in crowds (26). In this study, the scores of obsessive-compulsive symptoms in RA patients were higher than those in OA patients, but they did not meet the diagnostic criteria of obsessive-compulsive disorder. The main manifestations were repeated checking of joint status (such as frequent confirmation of joint swelling or pain), cleaning behavior (such as excessive hand-washing or disinfection to avoid infection), and symmetry/order obsessive-compulsive thoughts (such as adherence to a fixed sequence of medication or activities). As a result, patients are prone to engage in compulsive behavior (27).

The experiential avoidance scores in the two groups showed that the total AAQ-II score in patients with RA was significantly higher than in those with OA. Acceptance and commitment therapy (ACT) is a mindfulness-based cognitive behavioral therapy that aims to improve patients’ psychological flexibility, reduce experiential avoidance, and promote mental health (28). Most patients with OA and RA have insufficient understanding of their diseases, lack channels for disease-related knowledge, and are full of concerns about treatment methods, surgical safety, and prognosis. Long-term joint pain leads to a decline in quality of life. These factors can cause varying degrees of negative emotions such as anxiety and depression (29, 30). The higher the AAQ-II score, the higher the experiential avoidance; that is, the lower the psychological flexibility. In this study, the total AAQ-II score of RA patients was higher than that of OA patients, indicating that RA patients’ acceptance of the disease was lower than that of OA patients. This is related to the prolonged course of RA, high disability rate, relatively expensive treatment costs, and the current lack of a radical cure for RA (31). However, current domestic and international research mainly focuses on ACT interventions, mostly for OA patients or patients undergoing total knee arthroplasty (32), with few ACT interventions for RA patients. Therefore, future interventions could be developed for RA patients to promote their mental health.

The total SRF-S score in RA patients was higher than that in OA patients, indicating that RA patients’ self-regulatory fatigue ability was lower than that of OA patients. This may be related to the continuous depletion of patients’ self-regulatory resources, leading to impaired cognitive, emotional, and behavioral regulation. The total SRF-S score of RA patients in this study was higher than the value (43.64 ± 9.55) reported by Zhou et al. (33), while the score for OA patients was lower. Nevertheless, both groups showed a moderate degree of self-fatigue, and the more severe the SRF, the worse their self-management behavior. Therefore, there is an urgent need for intervention research on SRF in both patient groups.

The SCL-90 score was used as the overall indicator for evaluating mental health status, so only its influencing factors were analyzed. The results showed that in the OA group, female gender, presence of comorbidities, AAQ-II, and SRF-S were influencing factors of SCL-90. In the RA group, female gender, disease duration≥5 years, AAQ-II, and SRF-S were the main factors influencing. In both groups of patients, female gender, AAQ-II, and SRF-S were common influencing factors of SCL-90.

The total SCL-90 score in female patients was higher than in male patients, which may be due to the different social roles and responsibilities assumed by men and women. Most women balance careers and families, thus facing greater pressure. Moreover, women are more sensitive and prone to psychological problems. Research shows that patients with chronic diseases experiencing multiple symptoms are more likely to experience self-regulatory fatigue (34). This paper indicates that patients with more prominent self-regulatory fatigue and lower psychological flexibility have more severe symptom distress. This may be related to the gradual decline in joint function in patients, as well as the accompanying symptoms of chronic diseases, such as joint pain, swelling, morning stiffness, and even joint deformity, which lead to a gradual decline in patients’ mental health. There is a statistically significant difference in the total SCL-90 score among RA patients with different disease durations, and patients with shorter disease durations have lower total SCL-90 scores, suggesting that longer disease duration is associated with a decline in patients’ mental health. The two groups of patients not only endure great physical pain but also suffer psychological torment, which brings an unbearable psychological burden and triggers psychological problems. If proper and effective measures are not taken, this will undoubtedly lead to a decrease in patients’ mental health.

In summary, this study demonstrated that both RA and OA patients experience significant psychological distress, with RA patients exhibiting higher SCL-90 scores than OA patients, especially in somatization, forced symptoms, and interpersonal sensitivity factors; these aspects, depression, anxiety, and terror, were more significant. Univariate and multivariable analyses consistently identified comorbidities as a robust predictor of psychological symptoms in the OA group, while disease duration emerged as the most significant factor in the RA group. Moreover, self-regulatory fatigue (SRF-S) and experiential avoidance (AAQ-II) were independently associated with SCL-90 scores in both groups, suggesting that these psychological processes may be important therapeutic targets.

Several limitations of this study should be acknowledged. First, the cross-sectional design precludes causal inference regarding the observed associations between disease-related factors and psychological distress. Second, the convenience sampling from a single tertiary hospital in Shandong Province limits the generalizability of our findings to broader populations. Third, disease activity measures (e.g., DAS28 for RA) and pain severity scores were not collected, potentially confounding the relationship between diagnosis and mental health outcomes. Finally, the SCL-90, while widely used, was developed several decades ago; future studies may benefit from incorporating more contemporary instruments such as the PHQ-9 and GAD-7 alongside multidimensional scales to enable cross-instrument validation.

In conclusion, psychological distress is a significant concern in both RA and OA patients, influenced by comorbidities, disease duration, self-regulatory fatigue, and experiential avoidance. These findings emphasize the need for comprehensive, biopsychosocial approaches to the management of rheumatic diseases that address both physical and mental health needs.

## Data Availability

The raw data supporting the conclusions of this article will be made available by the authors, without undue reservation.
