# Supplementary material for: A comparative study on mental health outcomes in patients with rheumatoid arthritis and knee osteoarthritis
Source: Front Med (Lausanne). 2026 Sep 3;13:1879435. doi: 10.3389/fmed.2026.1879435 (PMC13581939; doi:10.3389/fmed.2026.1879435)
Supplement: Supplementary file 1 [file Data_Sheet_1.PDF]

Supplementary Table S1. Bonferroni post-hoc pairwise comparisons for significant ANOVA results

Group	Dependent variable	Factor	Comparison	Mean difference	SE	*P*	P_{Bonferroni}
OA	SCL-90	Course of disease	5–9 years vs. <5 years	4.866	3.407	0.465	1.000
OA	SCL-90	Course of disease	≥10 years vs. <5 years	7.378	3.487	0.108	0.324
OA	SCL-90	Course of disease	≥10 years vs. 5–9 years	2.512	1.164	0.097	0.291
OA	SCL-90	Educational level	Secondary vs Primary	-1.384	1.161	-	-
OA	SCL-90	Educational level	Tertiary vs Primary	4.074	1.520	0.049	0.294
OA	SCL-90	Educational level	Other vs Primary	0.360	3.407	-	-
OA	SCL-90	Educational level	Secondary vs Tertiary	-5.458	1.461	0.002	0.012*
OA	SCL-90	Educational level	Secondary vs Other	-1.743	3.381	-	-
OA	SCL-90	Educational level	Tertiary vs Other	3.714	3.521	-	-
OA	SCL-90	Comorbidities	1–2 vs. none	6.545	0.795	<0.001	<0.001*
OA	SCL-90	Comorbidities	≥3 vs. none	14.542	1.025	<0.001	<0.001*
OA	SCL-90	Comorbidities	≥3 vs. 1–2	7.997	0.968	<0.001	<0.001*
OA	AAQ-II	Comorbidities	1–2 vs. none	0.691	1.484	-	-
OA	AAQ-II	Comorbidities	≥3 vs. none	3.950	1.627	0.049	0.147
OA	AAQ-II	Comorbidities	≥3 vs. 1–2	3.258	1.180	0.019	0.057
OA	SRF-S	Comorbidities	1–2 vs. none	1.571	1.213	0.591	1.000
OA	SRF-S	Comorbidities	≥3 vs. none	7.936	1.330	<0.001	<0.001*
OA	SRF-S	Comorbidities	≥3 vs. 1–2	6.365	0.965	<0.001	<0.001*
RA	SCL-90	Course of disease	5–9 years vs. <5 years	4.693	1.134	<0.001	<0.001*
RA	SCL-90	Course of disease	≥10 years vs. <5 years	9.024	1.652	<0.001	<0.001*
RA	SCL-90	Course of disease	≥10 years vs. 5–9 years	4.331	1.421	0.008	0.024*

AAQ-II: Acceptance and Action Questionnaire-II; SRF-S: Self-Regulatory Fatigue Scale.

*Bonferroni correction was applied within each set of comparisons for each dependent variable: Course of disease (3 comparisons, threshold α = 0.05/3 = 0.0167), Educational level (6 comparisons, threshold α = 0.05/6 = 0.0083), Comorbidities (3 comparisons, threshold α = 0.05/3 = 0.0167).

* Statistically significant after Bonferroni correction (P_{Bonferroni} < 0.05)
